# How Adolescent Subjective Health and Satisfaction with Weight and Body Shape Are Related to Participation in Sports

**DOI:** 10.1155/2014/851932

**Published:** 2014-06-12

**Authors:** Åse Eriksen Dyremyhr, Esperanza Diaz, Eivind Meland

**Affiliations:** Department of Global Public Health and Primary Care, Research Group of General Practice, Kalfarveien 31, 5018 Bergen, Norway

## Abstract

*Background*. Physical exercise has positive effects on health. However, its associations with self-rated health and body image, which are important predictors for adolescents' wellbeing and later morbidity, are complex. *Methods*. Cross-sectional survey among 2527 Norwegian adolescents. We examined the relations between self-reported gender, body size, amount and type of exercise and measures of self-rated health, drive for thinness, and desire to change body, with binary logistic regression analyses. *Results*. Girls and overweight students reported to a greater extent than their peers impaired self-rated health, weight concerns, and desire to change their body. Increasing amount of time spent on sports was related to improved self-rated health in a dose-response manner. Both girls and boys who engaged in individual sports with an advantage of leanness, but only girls engaged in team sports, reported an increased desire to change the body. However, weight concern was not related to amount or type of sports. *Conclusions*. Physical exercise is positively related to self-reported health but has negative associations with body image for many adolescents. Health promotion efforts should consider this paradox and stimulate physical activity and sports along with body acceptance.

## 1. Introduction

Physical exercise and sports have positive effects on physical and psychological health. Physical activity among adolescents is correlated with increased cardiovascular fitness and bone strength [1, 2] and inversely associated with insulin resistance, metabolic syndrome, and premenopausal breast cancer [3–6].

The psychological effects of physical activity are associated with reduced depression and reduced risk of hopelessness and suicidality amongst adolescents [7, 8]. Also, when exercise is based on motivational factors such as health, social participation, or stress management, it seems to decrease body dissatisfaction [9].

However, negative effects have also been associated with physical exercise. Extensive exercise is related to body dissatisfaction, especially amongst women with eating pathology [10, 11], and physical activity often results in increased focus and awareness concerning weight and shape [12]. Perceived sociocultural pressure and body dissatisfaction are especially associated with a compelling need to exercise [13].

Self-rated health (SRH) is an individual's subjective perception of his or her own health status and constitutes an essential predictive indicator for later morbidity, mortality, school-dropout, use of health care services, and social welfare [14, 15]. SRH is, however, dependent upon personal and value-dependent interpretations [16]. SRH is constructed early in life when parental influence is strong and remains relatively stable and insensitive of transient diseases during adolescence [17]. Poor self-rated health is positively associated with body dissatisfaction and negatively associated with exercise and school achievements [18, 19].

Body dissatisfaction represents the discrepancy between an individual's current and ideal body size and shape, and its prevalence increases throughout adolescence [20–22]. Some studies claim that body and weight dissatisfaction represent worse health threats than obesity in itself, as they may trap adolescents in a vicious cycle of futile dieting, health compromising weight shifts, dysfunctional motivation for exercise, and thereby increased vulnerability for modern ideals of body size and shape [23, 24].

However, most of the previously referred studies have focused on females and on how sports might contribute to the development of eating pathology. Although sports engagement is positively related to SRH, our hypothesis is that exercise might also be associated with negative health effects among adolescents in the general population. Thus, in a nonselected adolescent population, we aimed to study the distribution of self-rated health and body dissatisfaction as expressed by both drive for thinness and desire to change the body and to explore the association between these factors and sports' type and amount in both genders. We also aimed at exploring how these factors were related to body size.

## 2. Method

### 2.1. Participants

The study was based on data from three cross-sectional surveys carried out in 2001, 2005, and 2009 amongst tertiary school students in Førde in the county of Sogn and Fjordane, in Norway. All four schools in the city of Førde were chosen. Three of them offered vocational training, while the remaining school offered an academic curriculum. Students completed the questionnaires without other information than a brief instruction from the teacher followed by 45 minutes to complete the questionnaire.

The 2001, 2005, and 2009 surveys included 837 (50% females), 776 (49% females), and 914 (55% females) students, with response rates of 93, 87, and 90 percent, respectively. Missing responses were almost exclusively due to absence from school on the days of surveys. Students that were nonresponders on single variables were included in the analysis of remaining responded variables. The variables of interest were equivalent in each survey and the samples included the same schools at the three different years of study. As no other differences could be detected between the surveys and in order to increase statistical power of the study, the three study samples were combined.

### 2.2. Measures

In addition to self-reported information on gender, height, and weight, five other questions from the survey regarding sports activity, SRH, and body dissatisfaction were included in this study. The questions were based on the World Health Organization cross-national survey, health behaviour in school-aged children, which aims to increase the knowledge about lifestyle and health in adolescents [25]. These self-reported variables have proved reliable and valid among slightly younger age groups [19, 25], and also among adolescents at similar age [26]. SRH was assessed using the question “How healthy do you think you are?” with the response alternatives “very healthy,” “quite healthy,” and “not very healthy.” The number of categories was reduced, by combining “very healthy” and “quite healthy.”

Body dissatisfaction was measured using two variables. The first one was “Are you on a diet to lose weight?” with corresponding responses “Yes,” “No, but I need to lose weight,” and “No, because my weight is fine.” The categories “Yes” and “No, but I need to lose weight” were combined, thus respecting the underlying drive for thinness. The second question was “Is there anything about your body you would like to change?” with the response options “Yes” and “No” [25].

The sample was differentiated according to amount of time spent on physical activity during a week and type of sports performed. The latter was classified by whether the main activity was team sports, individual sports without advantage of leanness (e.g., horseback riding, snowboarding, tennis, and boxing), or individual sports with advantage of leanness (e.g., ballet, taekwondo, swimming, fitness, and running). The classification of leanness and nonleanness sports was based on a study about dieting habits in adolescent elite athletes [27]. Leanness and weight were not considered as important for performance of any of the team sports, which were consequently not further subclassified. The group that was not performing any kind of physical activity or sports during the week was the reference for both amount and type of sports.

### 2.3. Statistical Analysis

In the analyses, self-rated health and the two measures of body image (drive for thinness and desire to change the body) were the dependent variables. The groups classified by amount of exercise and qualities of sports, gender, age, and BMI were the independent variables, and these were treated as categorical. Stratification was used to explore the sample and its nature. The relationships between the independent variables and SRH and the two body image variables were tested with binary logistic regression and were presented as odds ratios (OR) with 95% confidence intervals (CIs). In the adjusted, full model analyses, gender, age, and categorical BMI were included together with the exercise amount and the sports type variables. We checked if gender specific interactions were evident by performing gender stratified analyses. The results were reported in gender separate tables only if CIs were not vastly overlapping. Only the relation between sports type and the desire to change body was gender specific. We also examined if the prevalence of the outcomes differed or if the gender associations with the outcomes differed across study years. The prevalence of outcomes was almost identical during the period 2001–2009. Small differences with overlapping CIs were observed across study years on the gender associations with the outcomes. We did not further explore interaction effects of time, as this was not the prime focus of the study. All the analyses were performed in SPSS version 18.0.

### 2.4. Ethics

The survey was anonymous. Students were also informed that participation was voluntary. The data were collected in sealed envelopes and registered and stored anonymously. The 2009 study was presented for the Regional Committee for Medical and Health Research Ethics in Health Region 3. However, owing to its anonymous nature, it was consented and omitted from any further ethical evaluation. The Norwegian Data Inspectorate accepted all three surveys.

## 3. Results

The population and the frequencies of the reported variables are presented in Table 1. Most of the students perceived themselves as healthy, boys more frequently than girls. Girls reported desire to change the body and weight concerns more frequently than boys. The majority reported some kind of physical activity, but 36% of the girls and 29% of the boys devoted no more than one hour per week to physical activity. A majority of the students spent a moderate to a great amount of time on sports. Participation in individual sports was more common among girls, while boys were more active in team sports.

Tables 2 to 4((a) and (b)) summarize both unadjusted and adjusted results of the logistic regression analyses of, respectively, SRH, drive for thinness, and desire to change the body according to amount of time spent on physical activity and type of sports.

A strong association between SRH and time spent on physical activity was revealed, as illustrated in Table 2. The dose-response association was evident. Girls and adolescents with deviating body size reported impaired self-rated health to a greater extent than their peers. All weight groups reported impaired SRH as compared with the slim weight group (10th percentile to the median group).

Table 3 shows that the risk of dieting or desire to lose weight was strongly gender and BMI dependent. Weight dissatisfaction was not associated with increasing time spent on physical activity. Performing team sports was protective for dieting or desire to lose weight in the unadjusted analyses, but this association disappeared in the adjusted analyses. Surprisingly, adolescents engaged in sports with an advantage of leanness were not significantly more weight concerned than their nonsporting peers. We performed supplementary, stratified analyses (not shown) and were unable to reveal any gender specific interactions on these relations. However, weight dissatisfaction increased conspicuously even with modest overweight (BMI from 22.8 to 26.4) and escalated to the extreme among adolescents with BMI > 26.5.

Tables 4((a) and (b)) illustrate that the desire to change the body was also strongly affected by BMI. The OR that girls reported desire to change body was 5.10 (4.08–6.37) in the full model (not shown). The gender separated tables reveal that this desire increased with age only among girls. Engaging in individual sports with advantage of leanness was associated with the desire to change the body in both genders. Team sports were associated with body dissatisfaction only among girls.

The mediation effects from BMI were negligible despite the fact that BMI impacted the result variables profoundly. However, we found a significant mediation effect between the sports variables. The differences that we found between the sports amount groups concerning weight and body dissatisfaction were accounted for by sports type. Thus, in the final models only students of both genders engaged in sports with leanness advantage and also girls engaged in team sports were more likely than others to report body dissatisfaction.

## 4. Discussion

### 4.1. Main Finding of This Study

We revealed a paradoxical association between adolescent health and sports: increasing amount of sports is correlated with good self-rated health on the one hand, but engagement in individual sports with advantage of leanness and also team sports among girls are related to body dissatisfaction on the other hand.

### 4.2. Strengths and Limitations of This Study

Our study has several strengths. The response rate was very high and the survey embraced all vocational and academic students in the municipality of Førde. Students in tertiary school in the county of Sogn and Fjordane move to Førde in order to study. The population is thus representative for both rural and urban Norwegian adolescents and probably adolescents in industrial countries worldwide as well. Inclusion of a vast diversity of sports activities and different measures of body image respect the nuances within the adolescent health.

However, cross-sectional studies cannot explain the direction of causality and also imply the possibility of misclassification and self-report bias. Considering that most individual sports tend to have leanness as a potential advantage, only a small proportion of the students engaged in sports where leanness was not regarded as beneficial. Therefore, this group showed broad confidence intervals and weak associations. It should also be noted that the classification of types of sports after the main activity was rather deterministic, as many were active in both team sports and individual sports with and without leanness requirements.

The full model analyses adjusted to the interrelationship between sports measures and other possible confounders. We used categorical BMI and thus the J-curve association with body image was respected. However, we cannot exclude other possible confounders that could influence our results. Home situation with respect to parental divorce, death or sickness in family, or poor economy has the potential to influence both the opportunity to participate in sports activities as well as SRH and body image.

The prevalence of the outcomes that we used varied from 13% to 75%. We should therefore not confuse ORs with prevalence ratios, especially concerning desire to change body, but also weight concern.

### 4.3. What Is Already Known on This Topic

In line with previous studies [14, 15], we confirmed that increasing amount of sports was strongly associated with positive SRH. A systematic review revealed that physical activity improved self-esteem and that especially team sport was associated with improved health outcomes [28]. This association was evident in the present study only in the unadjusted analysis.

Our study is in accordance with a previous study on adolescent girls showing that the majority participated in individual sports with advantage of leanness, and body dissatisfaction was more strongly associated with BMI than with the nature of sports [29]. Interestingly, the girls in this study, who exercised irregularly, attempted to lose weight to a greater extent than those regularly exercising. This might be due to motivational factors, as demonstrated by a clinical study testing body image by means of digital photo distortion technique in which body image improved right after exercise [12]. Yet another study revealing a positive relationship between exercise and improved body image found that motivational factors partly explained this relationship and that appearance and weight motivation were associated with body dissatisfaction to a greater extent than fitness and health motivation [9]. In the same line, these previous publications indicate that the immediate alleviating effect of exercise on poor body image depends on type of motivation and personal trait. Studies linked to the self-determination theory also find that the quality of motivation both improves subjective health and is associated with sustained physical activity [24].

Regarding body dissatisfaction and physical exercise, a meta-analysis of 57 intervention studies showed that exercise improves body image [30]. A previous study on Norwegian adolescent elite athletes found no difference in dieting habits between the leanness and nonleanness sports athletes [27]. In accordance with this, body dissatisfaction related to sports in our study was not expressed with weight concern. Two previous studies amongst boys showed that body dissatisfaction and weight concerns were less prevalent amongst team sports participants [31] and football players compared to cross-country runners [32]. The same distinction is seen in our male population, but not amongst girls.

### 4.4. Implications

Impaired SRH is an independent predictor for later health and social problems [26]. In our study good SRH was associated with increasing amount of time spent on sports. This is in line with previous research, although the dose-response relation was not examined in earlier studies. This may have an important implication for health promotion in adolescence.

Although the odds for reporting body concern only modestly increased with leanness sports activities, the health consequences might be considerable due to the high prevalence of body concern. Why this concern depends on sports type may be explained by motivational differences. Also the gender difference concerning the association between team sports and body dissatisfaction might be explained by motivational differences. Such differences have been demonstrated in other motivational research among adults [33].

In western societies we emphasize the health compromising effects of overweight and obesity. Such a focus leads to prevalent body and weight dissatisfaction and preoccupation [19]. The stigmatization of oversized bodies may be accompanied by self-reproach and subjective health jeopardy, which may be more important factors explaining increased morbidity and mortality in obese populations than obesity in itself [23]. Therefore, it seems that we need a paradigmatic shift in the way we approach the obesity epidemic in western societies. Among adolescents we are also concerned that a one-dimensional focus on overweight and obesity may precipitate eating disorders. A unifying perspective for health promotion that emphasizes building of self-esteem and body satisfaction has therefore been suggested [34].

## 5. Conclusion

Physical exercise is positively related to self-reported health but has negative associations with body image for some adolescent groups. Health promotion efforts should consider this paradox and promote physical activity and sports along with body acceptance.

## Figures and Tables

**Table 1:** Age, BMI, sports habits, self-rated health, and body image variables according to gender.

Total 2510^1^	Girls (*n* = 1291)	Boys (*n* = 1219)
Age group, *n* (%)		
15–17 years	865 (67)	864 (71)
18–20 years	380 (29)	288 (24)
>20 years	46 (4)	67 (5)
Body mass index mean (SD)	22 (3.3)	23 (3.2)
Amount of physical activity during a week, *n* (%)		
“No”	167 (13)	158 (13)
“0.5–1 hour” (small amount)	298 (23)	197 (16)
“2–4 hours” (moderate amount)	458 (36)	384 (32)
“4 or more hours” (great amount)	362 (28)	471 (39)
Type of sports, *n* (%)		
Not sports	329 (27)	261 (23)
Team sports	238 (20)	377 (34)
Individual sports without advantage of leanness	77(6)	53 (5)
Individual sports with advantage of leanness	562 (47)	417 (38)
Self-rated health, *n* (%)		
“Very healthy” and “Quite healthy”	1085 (84)	1095 (90)
“Not very healthy”	207 (16)	119 (10)
On diet to lose weight, *n* (%)		
“No, weight fine”	663 (52)	963 (79)
“Yes" and “No, but need lose”	620 (48)	251 (21)
Desire to change body, *n* (%)		
“No”	166 (13)	510 (42)
“Yes”	1120 (87)	705 (58)

^1^17 out of a total of 2527 respondents did not answer the gender question.

**Table 2:** Poor self-rated health according to amount and type of sports.

Predictor variable	Univariate, unadjusted analysis OR (95% CI)	Adjusted, full model analysis OR (95% CI)
Reference category: gender boys		
Girls	1.76 (1.38–2.23)	1.70 (1.29–2.29)
Reference category: age group <18 years		
18–20 years	1.43 (1.11–1.84)	1.28 (0.66–2.47)
>20 years	1.07 (0.60–1.90)	1.70 (0.86–3.34)
Reference category: BMI category 10th percentile-median		
<10th percentile (<18.74)	2.22 (1.50–3.29)	1.68 (1.07–2.64)
Median-90th percentile (21.79–26.35)	1.30 (0.97–1.74)	1.54 (1.11–2.13)
>90th percentile (>26.46)	3.60 (2.51–5.16)	3.85 (2.55–5.79)
Reference category: no physical activity during a week		
Small amount	0.62 (0.45–0.87)	0.44 (0.28–0.68)
Moderate amount	0.36 (0.26–0.50)	0.30 (0.19–0.47)
Great amount	0.14 (0.09–0.20)	0.12 (0.07–0.21)
Reference category: no sports		
Team sports	0.35 (0.24–0.51)	1.04 (0.64–1.69)
Individual sports without advantage of leanness	0.70 (0.40–1.22)	1.50 (0.80–2.82)
Individual sports with advantage of leanness	0.63 (0.47–0.84)	1.28 (0.87–1.88)

**Table 3:** Dieting or desire to lose weight according to amount and type of sports.

Predictor variable	Univariate, unadjusted analysis OR (95% CI)	Adjusted, full model analysis OR (95% CI)
Reference category: gender boys		
Girls	3.59 (3.00–4.28)	6.58 (5.17–8.38)
Reference category: age group <18 years		
18–20 years	1.24 (1.03–1.50)	1.02 (0.81–1.29)
>20 years	1.05 (0.71–1.57)	0.70 (0.42–1.17)
Reference category: BMI category 10th percentile-median		
<10th percentile (<18.74)	0.80 (0.55–1.16)	0.66 (0.44–0.99)
Median-90th percentile (21.79–26.35)	2.64 (2.16–3.24)	4.30 (3.37–5.47)
>90th percentile (>26.46)	16.03 (11.24–22.86)	39.3 (25.3–61.0)
Reference category: no physical activity during a week		
Small amount	1.20 (0.90–1.60)	0.77 (0.50–1.19)
Moderate amount	1.11 (0.85–1.44)	0.83 (0.55–1.27)
Great amount	0.70 (0.53–0.92)	0.67 (0.43–1.04)
Reference category: no sports		
Team sports	0.58 (0.45–0.74)	0.84 (0.59–1.21)
Individual sports without advantage of leanness	0.91 (0.62–1.36)	0.87 (0.52–1.46)
Individual sports with advantage of leanness	1.09 (0.89–1.35)	1.22 (0.89–1.67)

**Table:** (a) Boys desire to change body according to amount and type of sports

Predictor variable	Univariate, unadjusted analysis OR (95% CI)	Adjusted, full model analysis OR (95% CI)
Reference category: age group <18 years		
18–20 years	0.92 (0.56–1.52)	1.14 (0.64–2.00)
>20 years	1.26 (0.74–2.17)	1.53 (0.84–2.81)
Reference category: BMI category 10th percentile-median		
<10th percentile (<18.74)	1.57 (0.98–2.52)	1.64 (0.97–2.77)
Median-90th percentile (21.79–26.35)	1.38 (1.07–1.79)	1.42 (1.08–1.86)
>90th percentile (>26.46)	3.83 (2.41–6.10)	3.86 (2.32–6.42)
Reference category: no physical activity during a week		
Small amount	1.37 (0.90–2.09)	1.01 (0.60–1.71)
Moderate amount	1.35 (0.93–1.95)	0.96 (0.58–1.58)
Great amount	1.52 (1.06–2.18)	1.23 (0.74–2.06)
Reference category: no sports		
Team sports	1.06 (0.78–1.46)	0.98 (0.65–1.47)
Individual sports without advantage of leanness	0.93 (0.51–1.67)	0.82 (0.43–1.55)
Individual sports with advantage of leanness	1.73 (1.26–2.37)	1.65 (1.12–2.45)

**Table:** (b) Girls desire to change body according to amount and type of sports

Predictor variable	Univariate, unadjusted analysis OR (95% CI)	Adjusted, full model analysis OR (95% CI)
Reference category: age group <18 years		
18–20 years	4.64 (2.48–8.69)	3.32 (1.64–6.73)
>20 years	5.01 (2.56–9.81)	3.53 (1.68–7.41)
Reference category: BMI category 10th percentile-median		
<10th percentile (<18.74)	0.94 (0.58–1.52)	0.95 (0.57–1.59)
Median-90th percentile (21.79–26.35)	1.87 (1.25–2.79)	2.05 (1.34–3.15)
>90th percentile (>26.46)	2.01 (0.98–4.13)	2.57 (1.17–5.65)
Reference category: no physical activity during a week		
Small amount	1.52 (0.91–2.54)	1.00 (0.54–1.86)
Moderate amount	2.12 (1.30–3.47)	1.51 (0.79–2.89)
Great amount	1.62 (0.99–2.66)	1.07 (0.53–2.15)
Reference category: no sports		
Team sports	2.29 (1.37–3.85)	1.98 (1.03–3.80)
Individual sports without advantage of leanness	0.88 (0.47–1.63)	0.68 (0.33–1.37)
Individual sports with advantage of leanness	2.14 (1.45–3.16)	1.70 (1.03–2.80)

## References

[1] Ortega FB, Ruiz JR, Hurtig-Wennlöf A, Sjöström M (2008). Physically active adolescents are more likely to have a healthier cardiovascular fitness level independently of their adiposity status. The European Youth Heart Study. *Revista Espanola de Cardiologia*.

[2] Tan VP, Macdonald HM, Kim S, Nettlefold L, Gabel L, Ashe MC (2014). Influence of physical activity on bone strength in children and adolescents: a systematic review and narrative synthesis. *Journal of Bone and Mineral Research*.

[3] Fedewa MV, Gist NH, Evans EM, Dishman RK (2014). Exercise and insulin resistance in youth: a meta-analysis. *Pediatrics*.

[4] Janssen I, Wong SL, Colley R, Tremblay MS (2013). The fractionalization of physical activity throughout the week is associated with the cardiometabolic health of children and youth. *BMC Public Health*.

[5] Countryman AJ, Saab PG, Llabre MM, Penedo FJ, McCalla JR, Schneiderman N (2013). Cardiometabolic risk in adolescents: associations with physical activity, fitness, and sleep. *Annals of Behavioral Medicine*.

[6] Boeke CE, Eliassen AH, Oh H, Spiegelman D, Willett WC, Tamimi RM (2014). Adolescent physical activity in relation to breast cancer risk. *Breast Cancer Research and Treatment*.

[7] Taliaferro LA, Rienzo BA, Miller MD, Pigg RM, Dodd VJ (2008). High school youth and suicide risk: exploring protection afforded through physical activity and sport participation. *Journal of School Health*.

[8] Brown HE, Pearson N, Braithwaite RE, Brown WJ, Biddle SJH (2013). Physical activity interventions and depression in children and adolescents: a systematic review and meta-analysis. *Sports Medicine*.

[9] LePage ML, Crowther JH (2010). The effects of exercise on body satisfaction and affect. *Body Image*.

[10] Davis C, Fox J, Cowles M, Hastings P, Schwass K (1990). The functional role of exercise in the development of weight and diet concerns in women. *Journal of Psychosomatic Research*.

[11] Thome J, Espelage DL (2004). Relations among exercise, coping, disordered eating, and psychological health among college students. *Eating Behaviors*.

[12] Vocks S, Hechler T, Rohrig S, Legenbauer T (2009). Effects of a physical exercise session on state body image: the influence of pre-experimental body dissatisfaction and concerns about weight and shape. *Psychology and Health*.

[13] White J, Halliwell E (2010). Examination of a sociocultural model of excessive exercise among male and female adolescents. *Body Image*.

[14] De Ridder KAA, Pape K, Johnsen R, Westin S, Holmen TL, Bjørngaard JH (2012). School dropout: a major public health challenge: a 10-year prospective study on medical and non-medical social insurance benefits in young adulthood, the Young-HUNT 1 study (Norway). *Journal of Epidemiology and Community Health*.

[15] Fylkesnes K, Forde OH (1991). The Tromso study: predictors of self-evaluated health—has society adopted the expanded health concept?. *Social Science and Medicine*.

[16] Idler EL, Benyamini Y (1997). Self-rated health and mortality: a review of twenty-seven community studies. *Journal of Health and Social Behavior*.

[17] Vingilis ER, Wade TJ, Seeley JS (2002). Predictors of adolescent self-rated health: analysis of the National Population Health Survey. *Canadian Journal of Public Health*.

[18] Mechanic D, Hansell S (1987). Adolescent competence, psychological well-being, and self-assessed physical health. *Journal of Health and Social Behavior*.

[19] Meland E, Haugland S, Breidablik H-J (2007). Body image and perceived health in adolescence. *Health Education Research*.

[20] Eisenberg ME, Neumark-Sztainer D, Paxton SJ (2006). Five-year change in body satisfaction among adolescents. *Journal of Psychosomatic Research*.

[21] Groesz LM, Blaivas JG, Levine MP, Murnen SK (2002). The effect of experimental presentation of thin media images on body satisfaction: a meta-analytic review. *International Journal of Eating Disorders*.

[22] Krones PG, Stice E, Batres C, Orjada K (2005). In vivo social comparison to a thin-ideal peer promotes body dissatisfaction: a randomized experiment. *International Journal of Eating Disorders*.

[23] Bacon L, Aphramor L (2011). Weight science: evaluating the evidence for a paradigm shift. *Nutrition Journal*.

[24] Gillison FB, Standage M, Skevington SM (2006). Relationships among adolescents’ weight perceptions, exercise goals, exercise motivation, quality of life and leisure-time exercise behaviour: a self-determination theory approach. *Health Education Research*.

[25] Currie C, Roberts C, Aea M (2004). *Young People’s Health in Context. Health Behaviour in School-Aged Children (HBSC) Study: International Report From the 2001/2002 Survey*.

[26] Breidablik H-J, Meland E, Lydersen S (2009). Self-rated health during adolescence: stability and predictors of change (Young-HUNT study, Norway). *European Journal of Public Health*.

[27] Martinsen M, Bratland-Sanda S, Eriksson AK, Sundgot-Borgen J (2010). Dieting to win or to be thin? A study of dieting and disordered eating among adolescent elite athletes and non-athlete controls. *British Journal of Sports Medicine*.

[28] Eime RM, Young JA, Harvey JT, Charity MJ, Payne WR (2013). A systematic review of the psychological and social benefits of participation in sport for children and adolescents: informing development of a conceptual model of health through sport. *International Journal of Behavioral Nutrition and Physical Activity*.

[29] Jankauskiene R, Kardelis K (2005). Body image and weight reduction attempts among adolescent girls involved in physical activity. *Medicina*.

[30] Campbell A, Hausenblas HA (2009). Effects of exercise interventions on body image: a meta-analysis. *Journal of Health Psychology*.

[31] Morano M, Colella D, Capranica L (2011). Body image, perceived and actual physical abilities in normal-weight and overweight boys involved in individual and team sports. *Journal of Sports Sciences*.

[32] McKay Parks PS, Read MH (1997). Adolescent male athletes: body image, diet, and exercise. *Adolescence*.

[33] Mildestvedt T, Meland E (2007). Examining the “Matthew Effect” on the motivation and ability to make lifestyle changes in 217 heart rehabilitation patients. *Scandinavian Journal of Public Health*.

[34] Neumark-Sztainer D (2005). Can we simultaneously work toward the prevention of obesity and eating disorders in children and adolescents?. *International Journal of Eating Disorders*.

